# A Hedonic Analysis of Processed Tomato Prices Using Italian Regional Markets Data

**DOI:** 10.3390/foods11060816

**Published:** 2022-03-12

**Authors:** Emilio De Meo, Gianluca Nardone, Francesco Bimbo, Domenico Carlucci

**Affiliations:** 1 Department of Agricultural and Environmental Sciences (DiSAAT), Università degli Studi di Bari Aldo Moro, Via Amendola 165/a, 70126 Bari, Italy; emilio.demeo@uniba.it (E.D.M.); domenico.carlucci@uniba.it (D.C.); 2 Department of Agricultural Sciences, Food, Natural Resources and Engineering (DAFNE), Università degli Studi di Foggia, Via Napoli, 25, 71122 Foggia, Italy; gianluca.nardone@unifg.it

**Keywords:** processed tomato, price variability, hedonic price model, implicit prices

## Abstract

The Italian market of processed tomatoes (whole peeled and unpeeled tomatoes, chopped tomato pulp, tomato purée, and concentrated tomato paste) rose, thanks to the Italian tradition of using such products in many recipes of cuisine, until early 2000; since then, it has declined to date. Moreover, such products are traditionally considered low-price products, and their market is characterized by intense price competition. Thus, recently, producers have started to differentiate their products as a way to achieve higher margins, and escape from competition in price. By using the sales data of Italian processed tomatoes sold in several retail stores and a hedonic price model, we estimated the implicit prices associated with several attributes that are currently available in processed tomato products on the market. We find that a protected designation of origin, organic certification, and flavoring, as well as the indication of tomato variety, are the most valuable features of processed tomato products sold in the Italian market. This implies that product differentiation strategies that could be suggested to producers as the most effective are those aimed at enhancing the territorial link of the product, the environmental sustainability of the production process, and organoleptic product features, as well as its convenience.

## 1. Introduction

Italy is the third largest producing country of processed tomatoes in the world, with a production of 5.16 million tons in 2020, after the U.S.A. (10.14 million tons), and China (5.8 million tons) [1,2]. Italian production of processed tomato products accounts for approximately 13.6% of global production, and 49% of European production, with a market size of approximately EUR 3.1 billion [3].

Italian tomato product processing is geographically concentrated in the Emilia-Romagna and Apulia regions, which account for 35% and 32% of national production, respectively [4]. Approximately 75% of tomatoes produced worldwide are earmarked for fresh consumption, while the remaining 25% are processed. This ratio largely varies across regions and geographical eating habits. In Italy, for instance, processed tomato is one of the main ingredients in national culinary traditions, widely used for pasta and pizza preparation [5].

The food industry processes tomatoes into four main product categories: whole tomatoes (peeled and unpeeled), chopped tomato pulp, tomato purée, and concentrated tomato paste. The production of these product categories requires that tomatoes undergo a rising degree of processing. Whole tomatoes (peeled and unpeeled) undergo minimal mechanical and thermal processes, while tomato purée and concentrated tomato paste undergo longer mechanical processes: the tomatoes are peeled, chopped, and cooked at higher temperatures and for longer amounts of time [6]. Among the various product categories, tomato purée is the most-used by Italian consumers, followed by tomato chopped pulp, and whole tomatoes [4,5].

Although Italians record one of the highest per capita rates of consumption of processed tomato products, the market for these products can be considered mature, and national consumption has recorded a progressive decline over the last 20 years. The consumption decline is largely due to consumers’ having less time available to cook and prepare meals at home [4,7]. Moreover, manufacturers face hurdles in increasing their margins since the processed tomato market is mainly characterized by competition in price between producers and retailers. Indeed, retailers compete to gain consumers by offering products at lower prices compared with those offered by their competitors [8,9]. Likewise, as a means to increase market share, manufacturers and retailers mostly focus on shrinking product price by lowering production costs, as well as by selling retailer brands (private labels) at a lower price compared to analogous branded products. Besides that, companies fiercely compete in pricing in foreign markets where they export their products [8,9,10].

In order to increase their margins, tomato processors could aim to reduce production costs by increasing plant efficiency, as previously reported by Čechura, Žáková Kroupová, and Samoggia [11], or attempt to differentiate their products in order to achieve higher market prices.

Reviewing the literature on consumer studies published over the last 20 years, four studies have investigated consumers’ acceptance of and preferences for transformed tomato products, as well as the impact of product features (e.g., organic, package characteristics, etc.) on product acceptance and preference [12,13,14,15]. Those studies mostly used small samples of consumers, ranging from 50 to 250 individuals, recruited in several geographical settings (e.g., Israel, Italy, Chile, and the Netherlands), and employed a wide variety of qualitative and quantitative techniques, which range from focus groups to means–end analysis and choice-based analysis [12,13,14,15]. Researchers found that product features such as color, brand name, and organoleptic features (e.g., taste, odor, etc.) increase the consumers’ acceptance of and preferences for tomato purée and whole peeled tomatoes [14,15]. In addition, product packaging material (e.g., glass), information on the tomatoes’ country of origin, and whether the product has been obtained according to sustainable practices (e.g., organic) increase consumer acceptance and preference [12,14,15]. However, the studies discussed above are based on stated-preferences data, where respondents (consumers) were typically asked to state their attitudes, perceptions, and intentions toward a product that has certain characteristics, as well as eliciting consumer preferences for product features via choice-based analysis. Thus, due to the subjective valuation of products, as well as the many metrics employed, one can argue that the high results variability across studies makes them not comparable, besides providing findings that can only apply to the consumers sampled. In addition, there is no insurance that what a person indicates in the focus group, interview, or choice experiment is what he or she would do in the real life [16].

Thus, in our analysis, we use the market price in order to have an objective measure of product attributes’ market performances; to this end, we employ a microeconomic model, the hedonic price model, in which the price evaluates the product attributes’ market success [17]. To the best of our knowledge, no study has assessed the implicit prices associated with processed tomatoes’ attributes as a means to support firms’ differentiation strategies, which may allow tomato processors to escape from price competition and receive fair revenue and higher profit. Indeed, over the last 10 years, manufacturers differentiated their products by introducing new product features, for instance, organic products, as well as marketing processed tomatoes that have grown in well-defined areas (e.g., 100% Italian, Protected Designation of Origin (PDO)), or that have a sweeter taste by using cherry tomato and datterino varieties [18,19,20,21].

Moreover, these investigations are common in other food markets, including yogurt [22,23], coffee [24], wine [25], extra-virgin olive oil [26], and fruit beverages [27]. Scholars using food product sales data and a hedonic price model consistently found that product features, such as the glass packaging and nutritional and health claims, as well as the organic label positively affect fruit beverages and yogurt prices on the Italian market, and can be used by producers to differentiate such products in the market. On the other hand, product price declines as the size of the package increases, as well as when the product is packaged using plastic material [22,23,27]. In addition, branded products are sold at higher prices compared to those sold under less-familiar or local brands; thus, investing in brand- and image-building activities is a marketing strategy that producers may pursue to differentiate their products [22,23,27]. Furthermore, a label indicating geographical origin, as well as one guaranteeing that the product has been produced according to standards (e.g., “fair trade label”) that guarantee fair revenues and working conditions to farmers, add value to agri-food products such as extra-virgin olive oil, wine, and coffee sold, respectively, in the Italian, U.K., and Swedish markets [24,25,26].

Therefore, measuring the market value of processed tomato features may offer valuable information to companies operating in a processed tomato market characterized by low profitability. To achieve our goal, we collected sales data (*n* = 729) of whole tomatoes (peeled and unpeeled), chopped tomato pulp, tomato purée, and concentrated tomato paste products sold in 16 stores (hypermarkets, supermarkets, minimarkets, and discounters) located in the South of Italy, in the province of Bari, and in the North, in the province of Turin.

We focused on the Italian market for two reasons: first, processed tomato sales account for more than half of total tomatoes sold in Italy due to the historical habit of Italian consumers of using processed tomato in many traditional recipes; second, the Italian tomato industry is highly concentrated; therefore, leading companies may have the technical know-how and financial resources to invest in product differentiation strategies. The remainder of this work is organized as follows: the next subsection describes the Italian tomato processing sector; Section 2 describes the data collected, as well as the theoretical and empirical model used; then, Section 3 discusses the empirical results, while Section 4 concludes the work by providing marketing recommendations for tomato processors, as well as avenues for future research.

### The Tomato Processing Sector in Italy

The Italian tomato processing industry has gone through a deep restructuring, reorganization, and modernization process since late 2000, when the Common Agricultural Policy reform drastically reduced public aid to tomato-growing farmers [27,28]. Lower public support for tomato producers led to a rise in the number and size of Producers Organizations (POs) in an attempt to concentrate tomato supply and increase the bargaining power with the industrial sector. The tomato processing sector, in turn, recorded several acquisitions and mergers of companies in an attempt to benefit from economy of scale and higher efficiency. This has led to just over one hundred firms operating in the sector to date [4,29,30].

The tomato processing companies are mainly located in the Emilia-Romagna region, the northern tomato processing district, and in Campania, the southern tomato processing district [4,31]. These companies transform tomatoes produced in Italy, whose production is concentrated in the 10 regions listed in Table 1. The North Italian tomato district is geographically concentrated in the Po Valley and processes tomatoes annually produced in the Emilia-Romagna, Lombardy, Veneto, and Tuscany regions. The North Italian tomato district is also characterized by a governance system that guarantees a high level of horizontal and vertical cooperation and coordination between farmers, POs, and the processing industry, which, overall, fairly share the revenues from sales.

The North Italian tomato district hosts a few medium–large processing companies, mostly cooperatives, that source the raw material via POs. The leading company in the tomato processing sector, Mutti, is located in Parma and earns 30% of processed tomato sales in the national market, with a turnover of EUR 465 million [30]. Mutti has three processing factories: in Basilicanova di Parma, the largest; in Oliveto Citra; and in Collecchio. These factories are supplied by 685 local farmers and mostly produce tomato pulps, whole peeled tomatoes, and concentrated tomato paste [32]. The second player in the Italian tomato processing sector is Conserve Italia, a cooperative consortium located in Bologna that owns brands such as Valfrutta, Yoga, Derby Blue, Jolly Colombani, Cirio, and Juver. The Conserve Italia consortium associates over 14 thousand farmers; annually processes over 600 thousand tons of fruit and vegetables in 11 factories, 8 of which are located in North Italy; and generates an aggregate turnover of EUR 872 million [33]. The top two players in the sector, Mutti and Conserve Italia, together with Petti, located in Venturina Terme (Tuscany), control over half of the Italian market for processed tomatoes [34].

The South Italian tomato district processes the tomatoes that are produced in regions such as Apulia, Campania, Lazio, Basilicata, Calabria, and Sicily. This district is characterized by a high number of processing companies, which are smaller than those in the North district, as well as poorly coordinated with local POs supplying the raw material [35,36]. These companies, such as Divella, Dentamaro, Desantis, and Granoro, record lower market performances compared to those located in the North of Italy and largely supply the southern Italian market, mostly at the regional level. Further, the companies’ market shares have recently been threatened by private labels, which have gained a market share of over 20% in value in the domestic market [34].

## 2. Materials and Methods

### 2.1. Data Collection and Description

Data collection was divided into two parts. The first part involved the selection of hypermarkets, supermarkets, minimarkets, and discounters located in the Apulia (province of Bari) and Piedmont (province of Turin) regions, from which we retrieved information on the characteristics of processed tomato products sold at the retail level. The sample of stores inspected included at least one of each type of selected outlet (hypermarkets, supermarkets, minimarkets, and discounters) within the two selected regions, for a total of 16 points-of-sale inspected, equally divided between the Apulia and Piedmont regions. The sampled stores were a representative sample of food stores for the provinces of Bari and Turin. In the second part, the selected stores were directly inspected by researchers, who collected the prices and product characteristics on all the references to processed tomato products available on the dedicated shelves. Data collection ran between June 2018 and February 2019, and product characteristics such as package size and material were collected, as well as whether the products were whole peeled or unpeeled tomatoes, in pieces, purée, or paste. In addition, we collected information on whether the product was flavored, produced using tomatoes grown with minimal use of chemicals (e.g., organic or integrated agriculture), and whether the raw material was sourced in a defined region or certified as a protected designation of origin. Lastly, we collected information on the tomato varieties used, whether the product was sold by a leading brand, and whether the product was sold in a hypermarket, a supermarket, a minimarket, or a discounter. The product features collected are listed in Table 2, and the final database encompasses information on 729 observations. In addition, references offering a temporarily reduced price were excluded from the survey to avoid potential estimate bias.

The collected price data of products showed high variability, ranging from a minimum value of 0.63 EUR/Kg to a maximum of 12.07 EUR/Kg, with an average price of 2.33 EUR/Kg. However, almost 80% of the collected price data were below 4 EUR/Kg. In addition, the most frequently offered package size was 400 gr, made with tin or glass material. Tomato purées represented the largest processed tomato category offered in the inspected stores, comprising 41% of the products sampled, followed by peeled tomatoes in pieces and whole peeled tomatoes, representing, respectively, 25% and 20% of products on the shelves. On the other hand, whole peeled tomatoes and tomato pastes accounted for 7% of the sampled products. In addition, a minority of processed tomatoes in our sample, 9%, were flavored with basil or spices. Over half of the references included in the sample (56%) were obtained through a conventional manufacturing process; the remaining share encompassed products obtained through production methods with a lower environmental impact compared to conventional ones (e.g., integrated agriculture, sustainable use of water, low carbon footprint, etc.), or organic ones. In addition, 82% of the sampled products use Italian-grown raw material (tomato), indicated on the product’s label using different wording, such as, “100% Italian”, “only Italian tomato”, “tomato is grown in Italy”, “from fresh Italian tomato”, or “Italian tomatoes”. In a few cases, sampled products reported the region from which the raw material was sourced, such as Tuscany, Sicily, Apulia, or Emilia-Romagna, or whether the tomatoes were certified as Protected Designation of Origin (PDO) (San Marzano dell’Agro Sarnese-Nocerino and Piennolo del Vesuvio). Of the sampled products, 13% indicated on the label the tomato variety used, such as datterino tomato, cherry tomato, or other varieties (e.g., Coimbra, Ercole, San Marzano, etc.), having sweeter and less acidic taste compared to the conventional tomato. Lastly, 30% of the sampled products were sold under leading brands, such as Mutti, Cirio, and Valfrutta. On the other hand, 20% of processed tomatoes on the shelves were sold under private labels (e.g., Coop, Selex, Despar, Auchan, Carrefour, Il Gigante, Simply, Crai), while products with brands exclusively sold in the discount outlets (e.g., Italiamo, Gustato, Campo Largo, Cuore Mediterraneo, Cà dell’Orto, etc.) and other minor brands with a local diffusion (e.g., Petti, Divella, Desantis, Granoro, Agromonte, Dentamaro, La Fiammante, Rosso Gargano, De Cecco, La Torrente, La Paesana, Casar, etc.) jointly covered the remaining 50% of total references sampled.

### 2.2. Empirical Model and Statistical Analysis

To calculate the monetary value of a product’s characteristics, we employed the hedonic price model, rooted in the microeconomic theory and firstly proposed by Rosen in 1974. The hedonic price theory considers a product as a bundle of characteristics, and each consumer in the market purchases the product that encompasses the unique bundle of features that maximize his or her utility. Likewise, companies maximize profits by selecting a product’s price according to the attributes it contains, with the price increasing as the number of attributes included in the product increases. The product’s price on the market reflects the buyers’ marginal bid and sellers’ marginal offers, and the joint envelope of consumers’ bids and sellers’ offers generate the hedonic price function so that the price P of a product j can be described as [17]:

Pj = f(Zj) (1)

where Z is a vector of product features belonging to product j, while f(.) is an unspecified functional form. Equation (1) points out that the price consumers pay for product, P, is a function of the monetary values of each j attribute embedded in the Z product sold in the market, which can be calculated by partially differentiating (1) with respect to each attribute [17,37].

Moreover, since at market equilibrium, the marginal price a consumer pays for each attribute j corresponds to the marginal cost that the producer incurs in offering that attribute on the market, Equation 1 can be estimated via Ordinary Least-Squares (OLS). Furthermore, the implicit price of each product characteristic was calculated using the adjustment proposed by Kennedy et al. (1981) [38], given the dichotomic nature of the variables included in the model equation. The first column of Table 3 reports the variables included in the model; the second column reports the estimated parameters of Equation (1), using the price logarithmic transformation as the dependent variable along with standard errors in parentheses. The last column of Table 3 reports the implicit prices of each attribute (in percentage terms).

## 3. Results and Discussion

The results obtained through the hedonic price model suggest that processed tomato products can be considered highly differentiated food products. In addition, their market price is greatly affected by packaging features, brand, size, material, and the point-of-sale where the product is sold, as well as by intrinsic characteristics of the products. In particular, the estimation results from the hedonic price model provide a measure of the market value for these latter characteristics, the so-called implicit prices, that can be used to identify the main, profitable product differentiation strategies. Overall, we found that product differentiation strategies that could be suggested to producers as the most effective are those aimed at enhancing the territorial connotation of the product (PDO certification, domestic origin of tomato), the environmental sustainability of the production process (organic certification), the sensory preference for the product (use of special tomato varieties), and its convenience (preassembled tomatoes with spices, herbs, and aromatic plants).

In detail, the estimated parameters in Equation (1) are reported in Table 3, along with their standard errors in parentheses and the marginal prices of each attribute (in percentage terms), calculated using Kennedy’s (1981) adjustment. The baseline product is an unflavored tomato purée, sold in a glass bottle, with no indication of product origin, and produced using tomatoes grown by a conventional method. On the baseline product’s label, there is no indicated tomato variety, and the product was sold under a minor brand in a hypermarket at an average price of 2.33 EUR/kg. The model shows an adjusted R^2^ equal to 0.83 with a statistically significant value of the F-Statistic, indicating the joint significance of coefficient regressors.

Firstly, the findings in Table 3 show that package size and material are statistically significantly related to the price of processed tomato products. Larger packages (“*Ln_Format*”) are associated with lower retail prices, as the estimates associated with the logarithmic transformation of package size is equal to −0.453. Such a result suggests that, as the container size increases by one percent, the product price decreases by the same magnitude. Moreover, the estimated marginal prices for products sold in packages other than glass, such as tin (“*Tin*”), brick (“*Brick*”), or plastic or aluminum (“*Other*”), are sold with price discounts ranging from −16.28% to −27.06%, corresponding to a discount between −0.38 EUR/kg and −0.63 EUR/kg.

Thus, our findings suggest that tomato products sold in small glass packages are preferred by consumers in the market. Consumers often infer the product’s wholesomeness when it is packaged in glass, for which they are willing to pay a premium price [39]. In addition, glass-packaged products allow consumers to assess the product’s color and texture, generating taste expectations about the product, and glass bottles are traditionally used to store the products and largely preferred by consumers over other packaging materials [15,40]. Consumer preferences for smaller glass packages in also consistent with findings from other studies investigating the implicit prices of product attributes in other food markets, such as extra-virgin olive oil, yogurt, and wine [21,22]. However, the higher market price for glass-packaged tomato products may reflect the higher cost of glass compared to that of other materials [41].

Secondly, the estimated results related to the analyzed product categories point out that whole peeled and unpeeled tomatoes, tomatoes in pieces, and tomato paste record higher implicit prices compared to tomato purée products (baseline), which is the most traditional category of tomato derivatives widely used by Italians. Whole unpeeled tomatoes, whole peeled tomatoes, tomatoes in pieces, and tomato paste, are sold at premium prices of +25.05%, +10.05%, +16.79%, and +133.52%, respectively, compared to the baseline price. These premiums likely reflect consumer interest in product categories in which the raw material undergoes a milder thermal process, thereby preserving the sensorial features of fresh tomatoes. Therefore, these product categories are perceived as having a higher level of “freshness” and “naturalness”; thus, a price premium is attached to them [5,42]. However, the largest price premium attached to tomato concentrates (+133.52%) must be interpreted with great caution, as it may reflect the higher production cost, related to the intense thermal treatment required to remove most of the tomato’s water, compared to that of the other tomato categories. Moreover, consumers are shifting from highly concentrated products, such as tomato paste, to less-concentrated and higher-value-added products, such as tomato purée, tomatoes in pieces, and organic products [42].

Thirdly, the estimated results show that tomato products flavored (“*Flavored*”) with spices, herbs, and aromatic plants record positive and significant effects on processed tomato prices in Italy: +41.49%, or 0.96 EUR/kg, compared to the average product price. Such a premium is likely due to growing consumer interest in products characterized by a higher service content, such as preassembled agri-food products, which are easier to prepare and demand less time to be served [43].

Fourthly, our findings indicate that sustainable-related attributes added to tomato products, such as label information indicating that the raw material was produced according to organic (“*Organic*”) or integrated (“*Integrated*”) production standards, have a positive and statistically significant effect on the product’s price. Organic is the most valuable attribute on the market, with a premium of +43.16% or +1.00 EUR/kg, everything else remaining constant. On the other hand, products obtained from tomatoes produced according to integrated standards (e.g., limited chemical inputs) show positive premiums of +7.59%. In terms of monetary value, tomato products obtained using integrated practices add a premium of +0.177 EUR/kg. These results confirm the growing consumer interest in selecting agri-food products with a lower impact on the environment, such as those obtained using practices that limit the use of chemicals in their production process, over conventional ones, whose negative impact on the environment was previously confirmed by Becker et al. (2016), who studied the organic tomato consumer [12]. In addition, the selection of organic tomato products or ones obtained using integrated practices are considered tastier and healthier compared to conventional ones; thus, they are preferred in the market. This may additionally justify the premium attached to organic and integrated tomato products [13]. The market’s preference for organic products is also consistent with the results of research that found price premiums for the organic versions of extra-virgin olive oils and yogurts by using sales data and a hedonic price model [21,22,25].

Fifthly, the results show that, other characteristics being equal, the tomato variety indication on the label adds value to the product. In detail, the datterino variety indication (“*Datterino*”) is associated with a price premium equal to +43.41%, or approximately 1.01 EUR/kg; the cherry variety indication (“*Cherry*”) is associated with an implicit price of +38.22%, about 0.89 EUR/kg; and minor varieties (Coimbra, Ercole, San Marzano, etc.) (“*Other varieties*”) add a premium price of +35.17%, close to 0.82 EUR/kg. These results are likely related to the growing interest of Italian consumers in tomato products with the sensory characteristics of sweetness and a low level of acidity compared to the conventional tomato [4].

Sixthly, the origin of the raw material strongly affects the retail price of processed tomatoes. The Italian indication of raw material (“*Italian*”) shows, ceteris paribus, a positive and significant effect on the tomato product price of +13.46%, corresponding to a monetary value of about 0.31 EUR/kg. The price premium rises as the indication of raw-product origin refers to a smaller and more well-defined region, such as Apulia, Tuscany, Sicily, Emilia-Romagna, and other regions; in this case, the price premium rises and ranges from +23.74% to +69.84%, or approximately from +0.55 EUR/kg to 1.63 EUR/kg. The price premium is even higher for a product with a protected designation of origin (PDO) (e.g., PDO “Tomato of San Marzano dell’Agro Sarnese-Nocerino” or PDO “Pomodorino del Piennolo del Vesuvio”) that certifies the well-defined area in which the product was obtained; the increase in price premium is +130.99%, corresponding to a monetary value of 3.05 EUR/kg. These results are consistent with studies suggesting that Italians’ food choices are affected by a home bias, as Italians often prefer domestic products over foreign ones. The preference for Italian agri-food products over foreign analogs has been previously recorded in the main Italian agri-food markets, such the wine [44], extra-virgin olive oil [45,46], milk [47], and pasta [48]. Our estimates show that Italians attach a higher value to products obtained from tomatoes grown in historical tomato production regions. Moreover, in the transformed tomato products, the PDO geographical indication labels show a positive and significant effect on price. This attribute records the highest price premium among all the processed tomato attributes taken into account in our analysis, a result consistent with several studies that also found that consumers, including those in the Italian market, prefer products with a geographical indication over regular ones, and are willing to pay higher prices for such products [14,49,50,51]. However, the higher price of tomato products with geographical indications may reflect the higher cost of PDO products, since farmers and producers willing to sell their products with a PDO label have to meet costly production standards that are frequently regarded as a barrier to compliance with PDO standards [52].

Seventhly, estimates related to brand show that leading brands positively affect the prices of processed tomatoes compared to the minor brands used as a baseline. The brand names of leading companies, such as Mutti, Cirio, and Valfrutta, are associated with a price premium ranging from +16.09% for Valfrutta (0.37 EUR/kg) to +49.79% for Cirio (1.16 EUR/kg). Mutti is associated with a price premium of +40.79% (0.95 EUR/kg). These results are likely due to consumers’ higher confidence and acceptance of tomato products that are sold under a brand they are familiar with. The brand was also found to be one of the most relevant product attributes affecting consumers’ processed tomato choices among Italians [14,53]. On the other hand, discount brands are associated with a price discount of −17.04% (−0.39 EUR/kg), which reflects competitive strategies essentially based on low-price, everyday products. The relevance of a product’s brand is also consistent with findings from scholars who investigated the price of other agri-food products, such as extra-virgin olive oil, yogurt, and wine [21,22,24].

Eighthly, and lastly, the product price is affected by the format of the store in which the product is sold: we formally accounted for it by adding dummy variables indicating whether the product was sold in a hypermarket, supermarket, minimarket, or discounter. The estimated results confirmed that tomato products sold in discount stores are characterized by a lower price of −10.4%, or 0.24 EUR/kg, than those sold in hypermarkets, reflecting the lower management and assortment costs that characterize discount stores compared to other stores. No significant price differences were detected between products sold in supermarkets and hypermarkets. However, those sold in minimarkets are sold at a higher price level of + 7.04%, or 0.16 EUR/kg, which reflects, to some extent, the higher logistics costs of delivering the products to such points-of-sale, often located in central and very congested urban areas [54].

## 4. Conclusions

In spite of the market trends that record multiple mergers and acquisitions that attempt to raise production efficiency and lower production costs, our results show that a differentiation strategy may also be a profitable strategy to add value to the product. Overall, our estimates confirm findings from existing studies that assessed the premium prices associated with several agri-food product features. Those studies using a hedonic price model and data on wine, yogurt, and fruit beverage sales found the highest price premiums are associated with indications of product origin as well as organic labels [21,46]. In addition, consistent with previous research, our findings point out that the market prefers the product in glass packaging and with particular sensory features [12,13,14,21,25,26].

In detail, from our study, the product feature with the highest price premium is raw material bearing a Protected Designation of Origin (PDO) label. Thus, producers and farmers may raise their revenues by using certified tomatoes as raw material, as well as by indicating the Italian (or even the regional) origin of the raw material on the label. The latter differentiation strategy would be of great help for farmers and producers with limited economic resources, as the implicit prices associated with these features are considerable. The use of tomatoes grown in compliance with organic standards or through production processes that minimize the use of chemical inputs may also represent a valid differentiation tool for producers. An alternative strategy of product differentiation would be based on the use of tomato varieties, such as datterino tomato and cherry tomato, whose tastes are greatly preferred by Italian consumers, and a premium price is associated with such product features. Our findings also suggest that flavoring the product matters in determining a product’s price premium. Thus, adding herbs or aromatic herbs and services to the product may actually pay off, as manufacturers may benefit from higher prices.

Lastly, we find that most leading brands benefit from an additional price premium compared to those supported by less familiar ones. Therefore, firms investing in the development of products may need to invest first in building and sustaining their brand image as a means to increase their tomato products’ marginal price.

## 5. Suggestions for Future Research

In spite of our results’ usefulness to provide guidance to food manufacturers in deciding whether or not to invest in a differentiation strategy, our analysis shows some limitations that may be overcome through future follow-up studies.

Firstly, our results refer to the Italian market, for which we use data sampled in two regions. Thus, future research will be aimed at obtaining estimates by expanding the number of stores sampled in multiple Italian regions. Secondly, our estimates reflect the average contribution of a tomato product’s features on price, but they do not allow for non-linearities or other effects of the attributes on prices. Thus, future research will use detailed household-level purchase data, flexible models, and estimation techniques (e.g., quantile regression) to tackle such limitations. Thirdly, a comparison of premium prices and relative costs associated with each attribute would offer a better picture of the actual mark-up achieved by adopting alternative differentiation strategies. Fourthly, and lastly, our results do not provide insights on the role played by consumers’ heterogeneity in the process of price formation, as they were obtained using aggregate market-level data. Therefore, more in-depth analysis may be conducted to investigate consumer preferences and willing-to-pay-for processed tomatoes attributes that gain higher premium prices.

## Figures and Tables

**Table 1:** Main Italian tomato producing regions (value in 000 tons).

Region	2018	2019	2020	2021
Emilia-Romagna	1670.708	1655.576	1881.332	1841.859
Apulia	1554.814	1482.295	1478.295	1472.77
Lombardy	496.9766	458.9275	613.4846	579.4991
Campania	228.9714	237.8094	240.1794	235.6336
Lazio	133.55	132.7	132.925	138.38
Veneto	88.5025	90.718	120.2555	140.138
Basilicata	117.7758	115.2725	115.2725	116.9912
Calabria	113.781	109.6543	110.1828	121.56
Tuscany	116.007	95.961	96.7169	124.523
Sicily	71.7	70.95	71.75	130.2
ITALY	4811.955	4729.021	5198.574	5256.601

Source: Italian National Institute of Statistics—section agriculture [28].

**Table 2:** List of variables and summary statistics (729 observations).

Variables ^a^	Share of Observations (%)	Price (EUR/kg)			
		Min	Max	Average	S. D.
*Price*		0.63	12.07	2.33	1.71
**Packaging size**					
*<400 g*	21	1.20	12.07	4.53	2.16
*400 g*	37	0.88	5.48	1.83	0.76
*401–699 g*	20	0.64	10.52	1.97	1.34
*700–800 g*	20	0.70	3.87	1.48	0.58
*>800 g*	2	0.63	1.25	0.88	0.20
**Packaging material**					
*Glass*	44	0.69	10.52	2.45	1.60
*Tin*	49	0.74	12.07	2.03	1.40
*Brick*	3	0.63	1.83	1.04	0.30
*Others (e.g., plastic, aluminum)*	4	1.50	11.00	5.61	2.71
**Product category**					
*Whole unpeeled tomatoes*	7	1.23	10.52	2.20	1.49
*Whole peeled tomatoes*	20	0.74	7.11	1.69	1.09
*Tomatoes in pieces*	25	0.78	5.48	2.05	0.97
*Tomato puree*	41	0.63	8.34	2.20	1.44
*Tomato paste*	7	1.73	12.07	6.12	2.31
**Flavoring**					
*Present*	9	1.00	8.34	4.04	1.75
*Absent*	91	0.63	12.07	2.15	1.61
**Production method**					
*Conventional*	56	0.63	8.75	2.05	1.50
*Integrated*	36	0.70	12.07	2.58	1.86
*Biological*	8	1.29	10.52	3.18	1.93
**Product origin**					
*Protected Designation of Origin (PDO)*	1	2.23	10.52	4.01	2.53
*Italy*	82	0.63	12.07	2.16	1.64
*Tuscany*	3	1.27	7.11	3.06	1.61
*Sicily*	3	2.78	6.25	4.99	1.01
*Apulia*	3	0.75	5.93	1.87	1.25
*Emilia-Romagna*	3	1.41	8.34	3.90	1.56
*Other regions*	1	1.07	4.64	2.90	1.31
*No origin indication*	4	0.70	3.94	1.70	0.84
**Product variety**					
*No indication*	87	0.63	12.07	2.08	1.61
*Cherry tomato*	4	1.98	6.25	4.42	1.39
*Datterino*	6	1.63	7.11	3.95	1.70
*Other varieties*	3	2.23	5.93	3.67	0.84
**Brand**					
*Mutti*	14	1.36	12.07	3.75	2.20
*Cirio*	12	1.41	11.00	3.16	1.99
*Valfrutta*	4	0.95	2.61	1.81	0.50
*Private labels*	20	0.80	5.31	1.81	0.95
*Discount brands*	8	0.65	3.61	1.41	0.80
*Other minor brands*	42	0.63	10.52	2.07	1.55
**Point-of-sale**					
*Supermarket*	36	0.81	9.62	2.19	1.55
*Minimarket*	12	0.85	12.07	2.86	2.34
*Discounter*	9	0.65	4.57	1.44	0.84
*Hypermarket*	43	0.63	10.52	2.49	1.69

^a^ Adjustment made according to Kennedy (1981) for dummy variables.

**Table 3:** Estimated parameters and percentage premium price.

Variable	*β*	Percentage Premium Price ^a^
*Ln_Format*	−0.453 ***	−45.30%
	(0.036)	
**Packaging material**		
*Tin*	−0.263 ***	−23.10%
	(0.048)	
*Brick*	−0.316 ***	−27.06%
	(0.050)	
*Others (e.g., plastic, aluminum)*	−0.178 **	−16.28%
	(0.056)	
**Product category**		
*Whole unpeeled tomatoes*	0.224 ***	+25.05%
	(0.061)	
*Whole peeled tomatoes*	0.096 **	+10.05%
	(0.047)	
*Tomatoes in pieces*	0.155 **	+16.79%
	(0.040)	
*Tomato paste*	0.850 ***	+133.52%
	(0.057)	
**Flavoring**		
*Flavored*	0.346 ***	+41.39%
	(0.047)	
**Production method**		
*Integrated*	0.073 ***	+7.59%
	(0.026)	
*Organic*	0.360 ***	+43.16%
	(0.044)	
**Origin**		
*Protected Designation of Origin (PDO)*	0.855 ***	+130.99%
	(0.207)	
*Italy*	0.126 ***	+13.46%
	(0.044)	
*Tuscany*	0.507 ***	+65.87%
	(0.076)	
*Sicily*	0.533 ***	+69.84%
	(0.118)	
*Apulia*	0.214 **	+23.74%
	(0.088)	
*Emilia-Romagna*	0.511 ***	+66.14%
	(0.124)	
*Other regions*	0.346 **	+40.88%
	(0.137)	
**Variety**		
*Cherry tomato*	0.326 ***	+38.22%
	(0.115)	
*Datterino*	0.361 ***	+43.41%
	(0.047)	
*Other varieties*	0.305 **	+35.17%
	(0.151)	
**Brand**		
*Mutti*	0.342 ***	+40.79%
	(0.036)	
*Cirio*	0.401 ***	+49.25%
	(0.038)	
*Valfrutta*	0.150 **	+16.09
	(0.037)	
*Private labels*	−0.033	−3.24%
	(0.031)	
*Discount brands*	−0.187 ***	−17.04%
	(0.066)	
**Point-of-sale**		
*Supermarket*	−0.030	−2.90%
	(0.021)	
*Minimarket*	0.068 **	+7.04%
	(0.032)	
*Discounter*	−0.110 *	−10.40%
	(0.060)	
*Constant*	3.07 ***	
	(0.247)	
Number of Observation	729	
Adjusted R-squared	0.8360	
F (29,699)	178,6048	
*p*-value	0.000	

^a^ Adjustment made according to Kennedy (1981) for dummy variables. *, **, and *** are 10%, 5%, and 1% significance levels.

## Data Availability

The data presented in this study are available on request from the corresponding author. The data are not publicly available due to the type of authorization for their collection by store managers.
